# Supplementary material for: Identification of PANoptosis-associated genes in hepatic ischemia-reperfusion injury by integrated bioinformatics analysis and machine learning
Source: PLoS One. 2025 Dec 30;20(12):e0339651. doi: 10.1371/journal.pone.0339651 (PMC12752983; doi:10.1371/journal.pone.0339651)
Supplement: S1 Table — Seven hepatic IRI datasets (GSE151648, GSE12720, GSE15480, GSE23649, GSE87487, GSE112713, and GSE189539) were obtained from the Gene Expression Omnibus (GEO). (DOCX) [file pone.0339651.s001.docx]

**Table S1. Information of data applied to this study.**

| **GEO ID** | **Platform** | **Experiment type** | **Organism** | **Pre-transplantation** | **Post-transplantation** |
| --- | --- | --- | --- | --- | --- |
| GSE151648 | GPL21290 | High RNA-Seq | Homo sapiens | 40 | 40 |
| GSE12720 | GPL570 | Array | Homo sapiens | 13 | 13 |
| GSE15480 | [GPL6244](https://www.ncbi.nlm.nih.gov/geo/query/acc.cgi?acc=GPL6244) | Array | Homo sapiens | 6 | 6 |
| GSE23649 | GPL6947 | Array | Homo sapiens | 16 | 16 |
| GSE87487 | GPL11154 | High RNA-Seq | Homo sapiens | 10 | 10 |
| GSE112713 | GPL14951 | Array | Homo sapiens | 11 | 11 |
| GSE189539 | GPL24676 | single-cell RNA-seq | Homo sapiens | 4 | 4 |

Table 1.Basic information of data applied to this study

| **GEO ID** | **Platform** | **Experiment type** | **Organism** | **Pre-transplantation** | **Post-transplantation** |
| --- | --- | --- | --- | --- | --- |
| GSE151648 | GPL21290 | High RNA-Seq | Homo sapiens | 40 | 40 |
| GSE12720 | GPL570 | Array | Homo sapiens | 13 | 13 |
| GSE15480 | [GPL6244](https://www.ncbi.nlm.nih.gov/geo/query/acc.cgi?acc=GPL6244) | Array | Homo sapiens | 6 | 6 |
| GSE23649 | GPL6947 | Array | Homo sapiens | 16 | 16 |
| GSE87487 | GPL11154 | High RNA-Seq | Homo sapiens | 10 | 10 |
| GSE112713 | GPL14951 | Array | Homo sapiens | 11 | 11 |
| GSE189539 | GPL24676 | Single-cell RNA-seq | Homo sapiens | 4 | 4 |
