# Supplementary material for: Identification of PANoptosis-associated genes in hepatic ischemia-reperfusion injury by integrated bioinformatics analysis and machine learning
Source: PLoS One. 2025 Dec 30;20(12):e0339651. doi: 10.1371/journal.pone.0339651 (PMC12752983; doi:10.1371/journal.pone.0339651)
Supplement: S2 Table — A PANoptosis pathway gene set was established by integrating genes from cell death-related pathways in the Molecular Signatures Database (MSigDB). (DOCX) [file pone.0339651.s002.docx]

**Table S2. PANoptosis Gene Data**

| **KEGG_ NECROPTOSIS** | **REACTOME_PYROPTOSIS** | **KEGG_ APOPTOSIS** | **HALLMARK_APOPTOSIS** | **REACTOME_APOPTOSIS** | **PANoptosis gene set** |
| --- | --- | --- | --- | --- | --- |
| TNF | BAK1 | CASP10 | CASP3 | BAD | BAK1 |
| TNFRSF1A | TP63 | CASP9 | CASP9 | CFLAR | TP63 |
| TRADD | CHMP2B | CASP8 | DFFA | PSMB1 | CHMP2B |
| TRAF2 | BAX | CASP7 | CASP7 | PSMC4 | BAX |
| TRAF5 | GZMB | CHUK | CFLAR | BID | GZMB |
| RIPK1 | CHMP4B | PRKAR2B | BIRC3 | VIM | CHMP4B |
| BIRC2 | GSDMD | TNF | PMAIP1 | FAS | GSDMD |
| BIRC3 | GSDME | TNFSF10 | CASP8 | BAK1 | GSDME |
| XIAP | IL1A | BIRC3 | JUN | DAPK2 | IL1A |
| RBCK1 | CHMP3 | XIAP | BCL2L11 | CDH1 | CHMP3 |
| RNF31 | IRF1 | PPP3R2 | MCL1 | PSMA4 | IRF1 |
| SHARPIN | IL1B | PPP3CC | IL1B | DSG2 | IL1B |
| SPATA2L | CHMP2A | PPP3R1 | SPTAN1 | CASP8 | CHMP2A |
| SPATA2 | CASP1 | MYD88 | DIABLO | PRKCQ | CASP1 |
| CYLD | CASP5 | FADD | BAX | ROCK1 | CASP5 |
| FADD | TP53 | CFLAR | BIK | PSME4 | TP53 |
| CASP8 | CHMP7 | RIPK1 | IL1A | ARHGAP10 | CHMP7 |
| CFLAR | IL18 | BAD | BID | TP63 | IL18 |
| RIPK3 | CASP3 | IRAK4 | CDKN1A | TP73 | CASP3 |
| CYBB | CHMP4C | BID | GADD45A | PKP1 | CHMP4C |
| CAMK2A | IRF2 | BAX | DDIT3 | BAX | IRF2 |
| CAMK2D | CYCS | IKBKB | CDKN1B | PSMC5 | CYCS |
| CAMK2B | CHMP6 | CASP6 | TNF | ADD1 | CHMP6 |
| CAMK2G | HMGB1 | IL1A | GSN | DNM1L | HMGB1 |
| SLC25A4 | CASP4 | AKT1 | TNFSF10 | PPP1R13B | CASP4 |
| SLC25A5 | ELANE | CASP3 | CASP6 | DYNLL1 | ELANE |
| SLC25A6 | CHMP4A | AKT2 | SQSTM1 | PSME1 | CHMP4A |
| SLC25A31 |  | TNFRSF1A | FASLG | CLSPN | CASP9 |
| PPID |  | AKT3 | EGR3 | PSMD5 | DFFA |
| VDAC1 |  | CHP2 | CD44 | DSP | CASP7 |
| VDAC2 |  | ATM | FAS | PSMD8 | CFLAR |
| VDAC3 |  | ENDOG | IL18 | MAPK1 | BIRC3 |
| GLUD2 |  | NFKB1 | IGFBP6 | GZMB | PMAIP1 |
| GLUD1 |  | NFKBIA | PRF1 | PSMC6 | CASP8 |
| GLUL |  | CAPN2 | DAP | PSMA3 | JUN |
| PYGL |  | PIK3R5 | CCND1 | PSMC1 | BCL2L11 |
| PYGM |  | IKBKG | BTG3 | PSMB5 | MCL1 |
| PYGB |  | CAPN1 | F2R | ACIN1 | SPTAN1 |
| MAPK8 |  | IL3RA | SATB1 | PSMA6 | DIABLO |
| MAPK10 |  | IL3 | BNIP3L | PSME2 | BIK |
| MAPK9 |  | RELA | CASP4 | PSMA7 | BID |
| FTH1 |  | ENDOD1 | TNFRSF12A | E2F1 | CDKN1A |
| FTL |  | APAF1 | CREBBP | PSMD10 | GADD45A |
| PLA2G4E |  | PRKX | RHOB | XIAP | DDIT3 |
| PLA2G4A |  | CSF2RB | GPX3 | BMX | CDKN1B |
| JMJD7-PLA2G4B |  | TNFRSF10A | PDGFRB | STK24 | TNF |
| PLA2G4B |  | TRAF2 | TSPO | TRADD | GSN |
| PLA2G4C |  | TNFRSF10D | CCND2 | MAPK3 | TNFSF10 |
| PLA2G4D |  | NGF | XIAP | PSMD7 | CASP6 |
| PLA2G4F |  | TNFRSF10B | TIMP1 | TJP1 | SQSTM1 |
| ALOX15 |  | TNFRSF10C | CTNNB1 | BMF | FASLG |
| CAPN1 |  | MAP3K14 | IRF1 | GSDMD | EGR3 |
| CAPN2 |  | IL1RAP | HSPB1 | TNFRSF10A | CD44 |
| SMPD1 |  | IL1B | ADD1 | AKT2 | FAS |
| MLKL |  | IRAK2 | TIMP2 | BBC3 | IGFBP6 |
| PGAM5 |  | IL1R1 | BTG2 | CARD8 | PRF1 |
| DNM1L |  | IRAK1 | TIMP3 | GSDME | DAP |
| NLRP3 |  | TRADD | LEF1 | PSMA2 | CCND1 |
| PYCARD |  | PIK3R3 | CASP1 | MAPK8 | BTG3 |
| CASP1 |  | BCL2 | GPX1 | UNC5B | F2R |
| IL1B |  | BCL2L1 | BCL10 | PSMD3 | SATB1 |
| CHMP2A |  | BIRC2 | IGF2R | SEPTIN4 | BNIP3L |
| CHMP2B |  | IRAK3 | CDC25B | KPNB1 | TNFRSF12A |
| CHMP3 |  | PRKACA | AIFM3 | C1QBP | CREBBP |
| RNF103-CHMP3 |  | PRKACB | CD38 | PSMD11 | RHOB |
| CHMP4B |  | PRKACG | PPP3R1 | YWHAE | GPX3 |
| CHMP4A |  | PPP3CB | HGF | BIRC2 | PDGFRB |
| CHMP6 |  | TP53 | CLU | PSMD9 | TSPO |
| VPS4B |  | PPP3CA | ATF3 | LMNB1 | CCND2 |
| VPS4A |  | PIK3CA | LGALS3 | UNC5A | XIAP |
| CHMP1B |  | PIK3CB | LUM | KPNA1 | TIMP1 |
| CHMP1A |  | FAS | LMNA | TFDP2 | CTNNB1 |
| CHMP5 |  | DFFA | GADD45B | PSMD14 | HSPB1 |
| CHMP7 |  | CYCS | CDK2 | AKT3 | ADD1 |
| TRPM7 |  | DFFB | IFNB1 | FASLG | TIMP2 |
| IL1A |  | PIK3CD | RETSAT | TJP2 | BTG2 |
| IL33 |  | PRKAR1A | SMAD7 | APAF1 | TIMP3 |
| HMGB1 |  | FASLG | SOD1 | TNFRSF10B | LEF1 |
| TNFSF10 |  | PRKAR2A | PTK2 | PPP3CC | GPX1 |
| TNFRSF10A |  | PRKAR1B | ENO2 | TNFSF10 | BCL10 |
| TNFRSF10B |  | EXOG | HMOX1 | H1-3 | IGF2R |
| FASLG |  | PIK3CG | IER3 | H1-1 | CDC25B |
| FAS |  | AIFM1 | BCL2L10 | PSMF1 | AIFM3 |
| FAF1 |  | NTRK1 | CD2 | PSMB2 | CD38 |
| IFNA1 |  | PIK3R1 | GCH1 | TRAF2 | PPP3R1 |
| IFNA2 |  | PIK3R2 | MMP2 | TICAM1 | HGF |
| IFNA4 |  | CHP1 | VDAC2 | SEM1 | CLU |
| IFNA5 |  |  | TAP1 | YWHAH | ATF3 |
| IFNA6 |  |  | PLAT | PSMA1 | LGALS3 |
| IFNA7 |  |  | IFNGR1 | PSME3 | LUM |
| IFNA8 |  |  | APP | CASP9 | LMNA |
| IFNA10 |  |  | BRCA1 | YWHAQ | GADD45B |
| IFNA13 |  |  | ROCK1 | STK26 | CDK2 |
| IFNA14 |  |  | PSEN1 | DSG3 | IFNB1 |
| IFNA16 |  |  | DCN | DSG1 | RETSAT |
| IFNA17 |  |  | PSEN2 | APC | SMAD7 |
| IFNA21 |  |  | SOD2 | DBNL | SOD1 |
| IFNB1 |  |  | BMF | NMT1 | PTK2 |
| IFNG |  |  | EREG | TLR4 | ENO2 |
| IFNAR1 |  |  | KRT18 | PSMB7 | HMOX1 |
| IFNAR2 |  |  | TGFB2 | RIPK1 | IER3 |
| IFNGR1 |  |  | RELA | UACA | BCL2L10 |
| IFNGR2 |  |  | WEE1 | CASP6 | CD2 |
| JAK1 |  |  | RARA | TP53 | GCH1 |
| JAK2 |  |  | CD14 | PMAIP1 | MMP2 |
| JAK3 |  |  | CD69 | AKT1 | VDAC2 |
| TYK2 |  |  | PEA15 | PSMB6 | TAP1 |
| STAT1 |  |  | DNAJC3 | PSMA5 | PLAT |
| STAT2 |  |  | CASP2 | TP53BP2 | IFNGR1 |
| STAT3 |  |  | CTH | RPS27A | APP |
| STAT4 |  |  | PLCB2 | CDKN2A | BRCA1 |
| STAT5A |  |  | BMP2 | GSN | ROCK1 |
| STAT5B |  |  | HMGB2 | GAS2 | PSEN1 |
| STAT6 |  |  | PLPPR4 | APIP | DCN |
| IRF9 |  |  | H1-0 | UBC | PSEN2 |
| EIF2AK2 |  |  | TGFBR3 | BCL2L11 | SOD2 |
| TLR4 |  |  | EBP | LY96 | BMF |
| TICAM2 |  |  | TXNIP | PSMA8 | EREG |
| TICAM1 |  |  | ANKH | APPL1 | KRT18 |
| TLR3 |  |  | RHOT2 | PSMD4 | TGFB2 |
| ZBP1 |  |  | CYLD | PSMB4 | RELA |
| USP21 |  |  | GSTM1 | DFFA | WEE1 |
| SQSTM1 |  |  | GSR | LMNA | RARA |
| HSP90AA1 |  |  | BGN | PSMC2 | CD14 |
| HSP90AB1 |  |  | BCL2L1 | OMA1 | CD69 |
| TNFAIP3 |  |  | GNA15 | PSMD6 | PEA15 |
| PARP2 |  |  | MGMT | PRKCD | DNAJC3 |
| PARP3 |  |  | PPT1 | HMGB2 | CASP2 |
| PARP4 |  |  | F2 | CASP3 | CTH |
| BID |  |  | IL6 | YWHAZ | PLCB2 |
| BAX |  |  | SC5D | CASP7 | BMP2 |
| AIFM1 |  |  | IFITM3 | PSMC3 | HMGB2 |
| H2AX |  |  | RNASEL | YWHAB | PLPPR4 |
| H2AC20 |  |  | EMP1 | DAPK3 | H1-0 |
| H2AC12 |  |  | CAV1 | CTNNB1 | TGFBR3 |
| H2AC1 |  |  | DNM1L | FADD | EBP |
| H2AW |  |  | ANXA1 | H1-4 | TXNIP |
| H2AB3 |  |  | TOP2A | FNTA | ANKH |
| H2AC8 |  |  | ISG20 | STAT3 | RHOT2 |
| H2AC4 |  |  | SLC20A1 | PTK2 | CYLD |
| MACROH2A2 |  |  | MADD | DFFB | GSTM1 |
| MACROH2A1 |  |  | PPP2R5B | AVEN | GSR |
| H2AC19 |  |  | BCAP31 | YWHAG | BGN |
| H2AJ |  |  | ERBB3 | UBB | BCL2L1 |
| H2AB1 |  |  | NEDD9 | CD14 | GNA15 |
| H2AC17 |  |  | SAT1 | BCL2L1 | MGMT |
| H2AC18 |  |  | PDCD4 | BCL2 | PPT1 |
| H2AC11 |  |  | BCL2L2 | CYCS | F2 |
| H2AC21 |  |  | FEZ1 | PSMD1 | IL6 |
| H2AZ2 |  |  | ERBB2 | PSMD2 | SC5D |
| H2AC7 |  |  | DNAJA1 | SFN | IFITM3 |
| H2AZ1 |  |  | DAP3 | PLEC | RNASEL |
| H2AC15 |  |  | DPYD | MAGED1 | EMP1 |
| H2AC6 |  |  | NEFH | PAK2 | CAV1 |
| H2AC13 |  |  | PAK1 | SATB1 | DNM1L |
| H2AC14 |  |  | FDXR | DIABLO | ANXA1 |
| H2AC16 |  |  | GPX4 | H1-5 | TOP2A |
| H2AB2 |  |  | ETF1 | PSMD13 | ISG20 |
| PPIA |  |  | CCNA1 | BCAP31 | SLC20A1 |
| BCL2 |  |  | GUCY2D | MAPT | MADD |
|  |  |  | AVPR1A | DCC | PPP2R5B |
|  |  |  |  | H1-2 | BCAP31 |
|  |  |  |  | H1-0 | ERBB3 |
|  |  |  |  | HMGB1 | NEDD9 |
|  |  |  |  | DAPK1 | SAT1 |
|  |  |  |  | PSMD12 | PDCD4 |
|  |  |  |  | SPTAN1 | BCL2L2 |
|  |  |  |  | OCLN | FEZ1 |
|  |  |  |  | TFDP1 | ERBB2 |
|  |  |  |  | OPA1 | DNAJA1 |
|  |  |  |  | PSMB8 | DAP3 |
|  |  |  |  | PSMB10 | DPYD |
|  |  |  |  | PPP3R1 | NEFH |
|  |  |  |  | UBA52 | PAK1 |
|  |  |  |  | PSMB11 | FDXR |
|  |  |  |  | PSMB9 | GPX4 |
|  |  |  |  | TICAM2 | ETF1 |
|  |  |  |  | DYNLL2 | CCNA1 |
|  |  |  |  | PSMB3 | GUCY2D |
|  |  |  |  |  | AVPR1A |
|  |  |  |  |  | CASP10 |
|  |  |  |  |  | CHUK |
|  |  |  |  |  | PRKAR2B |
|  |  |  |  |  | PPP3R2 |
|  |  |  |  |  | PPP3CC |
|  |  |  |  |  | MYD88 |
|  |  |  |  |  | FADD |
|  |  |  |  |  | RIPK1 |
|  |  |  |  |  | BAD |
|  |  |  |  |  | IRAK4 |
|  |  |  |  |  | IKBKB |
|  |  |  |  |  | AKT1 |
|  |  |  |  |  | AKT2 |
|  |  |  |  |  | TNFRSF1A |
|  |  |  |  |  | AKT3 |
|  |  |  |  |  | CHP2 |
|  |  |  |  |  | ATM |
|  |  |  |  |  | ENDOG |
|  |  |  |  |  | NFKB1 |
|  |  |  |  |  | NFKBIA |
|  |  |  |  |  | CAPN2 |
|  |  |  |  |  | PIK3R5 |
|  |  |  |  |  | IKBKG |
|  |  |  |  |  | CAPN1 |
|  |  |  |  |  | IL3RA |
|  |  |  |  |  | IL3 |
|  |  |  |  |  | ENDOD1 |
|  |  |  |  |  | APAF1 |
|  |  |  |  |  | PRKX |
|  |  |  |  |  | CSF2RB |
|  |  |  |  |  | TNFRSF10A |
|  |  |  |  |  | TRAF2 |
|  |  |  |  |  | TNFRSF10D |
|  |  |  |  |  | NGF |
|  |  |  |  |  | TNFRSF10B |
|  |  |  |  |  | TNFRSF10C |
|  |  |  |  |  | MAP3K14 |
|  |  |  |  |  | IL1RAP |
|  |  |  |  |  | IRAK2 |
|  |  |  |  |  | IL1R1 |
|  |  |  |  |  | IRAK1 |
|  |  |  |  |  | TRADD |
|  |  |  |  |  | PIK3R3 |
|  |  |  |  |  | BCL2 |
|  |  |  |  |  | BIRC2 |
|  |  |  |  |  | IRAK3 |
|  |  |  |  |  | PRKACA |
|  |  |  |  |  | PRKACB |
|  |  |  |  |  | PRKACG |
|  |  |  |  |  | PPP3CB |
|  |  |  |  |  | PPP3CA |
|  |  |  |  |  | PIK3CA |
|  |  |  |  |  | PIK3CB |
|  |  |  |  |  | DFFB |
|  |  |  |  |  | PIK3CD |
|  |  |  |  |  | PRKAR1A |
|  |  |  |  |  | PRKAR2A |
|  |  |  |  |  | PRKAR1B |
|  |  |  |  |  | EXOG |
|  |  |  |  |  | PIK3CG |
|  |  |  |  |  | AIFM1 |
|  |  |  |  |  | NTRK1 |
|  |  |  |  |  | PIK3R1 |
|  |  |  |  |  | PIK3R2 |
|  |  |  |  |  | CHP1 |
|  |  |  |  |  | PSMB1 |
|  |  |  |  |  | PSMC4 |
|  |  |  |  |  | VIM |
|  |  |  |  |  | DAPK2 |
|  |  |  |  |  | CDH1 |
|  |  |  |  |  | PSMA4 |
|  |  |  |  |  | DSG2 |
|  |  |  |  |  | PRKCQ |
|  |  |  |  |  | PSME4 |
|  |  |  |  |  | ARHGAP10 |
|  |  |  |  |  | TP73 |
|  |  |  |  |  | PKP1 |
|  |  |  |  |  | PSMC5 |
|  |  |  |  |  | PPP1R13B |
|  |  |  |  |  | DYNLL1 |
|  |  |  |  |  | PSME1 |
|  |  |  |  |  | CLSPN |
|  |  |  |  |  | PSMD5 |
|  |  |  |  |  | DSP |
|  |  |  |  |  | PSMD8 |
|  |  |  |  |  | MAPK1 |
|  |  |  |  |  | PSMC6 |
|  |  |  |  |  | PSMA3 |
|  |  |  |  |  | PSMC1 |
|  |  |  |  |  | PSMB5 |
|  |  |  |  |  | ACIN1 |
|  |  |  |  |  | PSMA6 |
|  |  |  |  |  | PSME2 |
|  |  |  |  |  | PSMA7 |
|  |  |  |  |  | E2F1 |
|  |  |  |  |  | PSMD10 |
|  |  |  |  |  | BMX |
|  |  |  |  |  | STK24 |
|  |  |  |  |  | MAPK3 |
|  |  |  |  |  | PSMD7 |
|  |  |  |  |  | TJP1 |
|  |  |  |  |  | BBC3 |
|  |  |  |  |  | CARD8 |
|  |  |  |  |  | PSMA2 |
|  |  |  |  |  | MAPK8 |
|  |  |  |  |  | UNC5B |
|  |  |  |  |  | PSMD3 |
|  |  |  |  |  | SEPTIN4 |
|  |  |  |  |  | KPNB1 |
|  |  |  |  |  | C1QBP |
|  |  |  |  |  | PSMD11 |
|  |  |  |  |  | YWHAE |
|  |  |  |  |  | PSMD9 |
|  |  |  |  |  | LMNB1 |
|  |  |  |  |  | UNC5A |
|  |  |  |  |  | KPNA1 |
|  |  |  |  |  | TFDP2 |
|  |  |  |  |  | PSMD14 |
|  |  |  |  |  | TJP2 |
|  |  |  |  |  | H1-3 |
|  |  |  |  |  | H1-1 |
|  |  |  |  |  | PSMF1 |
|  |  |  |  |  | PSMB2 |
|  |  |  |  |  | TICAM1 |
|  |  |  |  |  | SEM1 |
|  |  |  |  |  | YWHAH |
|  |  |  |  |  | PSMA1 |
|  |  |  |  |  | PSME3 |
|  |  |  |  |  | YWHAQ |
|  |  |  |  |  | STK26 |
|  |  |  |  |  | DSG3 |
|  |  |  |  |  | DSG1 |
|  |  |  |  |  | APC |
|  |  |  |  |  | DBNL |
|  |  |  |  |  | NMT1 |
|  |  |  |  |  | TLR4 |
|  |  |  |  |  | PSMB7 |
|  |  |  |  |  | UACA |
|  |  |  |  |  | PSMB6 |
|  |  |  |  |  | PSMA5 |
|  |  |  |  |  | TP53BP2 |
|  |  |  |  |  | RPS27A |
|  |  |  |  |  | CDKN2A |
|  |  |  |  |  | GAS2 |
|  |  |  |  |  | APIP |
|  |  |  |  |  | UBC |
|  |  |  |  |  | LY96 |
|  |  |  |  |  | PSMA8 |
|  |  |  |  |  | APPL1 |
|  |  |  |  |  | PSMD4 |
|  |  |  |  |  | PSMB4 |
|  |  |  |  |  | PSMC2 |
|  |  |  |  |  | OMA1 |
|  |  |  |  |  | PSMD6 |
|  |  |  |  |  | PRKCD |
|  |  |  |  |  | YWHAZ |
|  |  |  |  |  | PSMC3 |
|  |  |  |  |  | YWHAB |
|  |  |  |  |  | DAPK3 |
|  |  |  |  |  | H1-4 |
|  |  |  |  |  | FNTA |
|  |  |  |  |  | STAT3 |
|  |  |  |  |  | AVEN |
|  |  |  |  |  | YWHAG |
|  |  |  |  |  | UBB |
|  |  |  |  |  | PSMD1 |
|  |  |  |  |  | PSMD2 |
|  |  |  |  |  | SFN |
|  |  |  |  |  | PLEC |
|  |  |  |  |  | MAGED1 |
|  |  |  |  |  | PAK2 |
|  |  |  |  |  | H1-5 |
|  |  |  |  |  | PSMD13 |
|  |  |  |  |  | MAPT |
|  |  |  |  |  | DCC |
|  |  |  |  |  | H1-2 |
|  |  |  |  |  | DAPK1 |
|  |  |  |  |  | PSMD12 |
|  |  |  |  |  | OCLN |
|  |  |  |  |  | TFDP1 |
|  |  |  |  |  | OPA1 |
|  |  |  |  |  | PSMB8 |
|  |  |  |  |  | PSMB10 |
|  |  |  |  |  | UBA52 |
|  |  |  |  |  | PSMB11 |
|  |  |  |  |  | PSMB9 |
|  |  |  |  |  | TICAM2 |
|  |  |  |  |  | DYNLL2 |
|  |  |  |  |  | PSMB3 |
|  |  |  |  |  | TRAF5 |
|  |  |  |  |  | RBCK1 |
|  |  |  |  |  | RNF31 |
|  |  |  |  |  | SHARPIN |
|  |  |  |  |  | SPATA2L |
|  |  |  |  |  | SPATA2 |
|  |  |  |  |  | RIPK3 |
|  |  |  |  |  | CYBB |
|  |  |  |  |  | CAMK2A |
|  |  |  |  |  | CAMK2D |
|  |  |  |  |  | CAMK2B |
|  |  |  |  |  | CAMK2G |
|  |  |  |  |  | SLC25A4 |
|  |  |  |  |  | SLC25A5 |
|  |  |  |  |  | SLC25A6 |
|  |  |  |  |  | SLC25A31 |
|  |  |  |  |  | PPID |
|  |  |  |  |  | VDAC1 |
|  |  |  |  |  | VDAC3 |
|  |  |  |  |  | GLUD2 |
|  |  |  |  |  | GLUD1 |
|  |  |  |  |  | GLUL |
|  |  |  |  |  | PYGL |
|  |  |  |  |  | PYGM |
|  |  |  |  |  | PYGB |
|  |  |  |  |  | MAPK10 |
|  |  |  |  |  | MAPK9 |
|  |  |  |  |  | FTH1 |
|  |  |  |  |  | FTL |
|  |  |  |  |  | PLA2G4E |
|  |  |  |  |  | PLA2G4A |
|  |  |  |  |  | JMJD7-PLA2G4B |
|  |  |  |  |  | PLA2G4B |
|  |  |  |  |  | PLA2G4C |
|  |  |  |  |  | PLA2G4D |
|  |  |  |  |  | PLA2G4F |
|  |  |  |  |  | ALOX15 |
|  |  |  |  |  | SMPD1 |
|  |  |  |  |  | MLKL |
|  |  |  |  |  | PGAM5 |
|  |  |  |  |  | NLRP3 |
|  |  |  |  |  | PYCARD |
|  |  |  |  |  | RNF103-CHMP3 |
|  |  |  |  |  | VPS4B |
|  |  |  |  |  | VPS4A |
|  |  |  |  |  | CHMP1B |
|  |  |  |  |  | CHMP1A |
|  |  |  |  |  | CHMP5 |
|  |  |  |  |  | TRPM7 |
|  |  |  |  |  | IL33 |
|  |  |  |  |  | FAF1 |
|  |  |  |  |  | IFNA1 |
|  |  |  |  |  | IFNA2 |
|  |  |  |  |  | IFNA4 |
|  |  |  |  |  | IFNA5 |
|  |  |  |  |  | IFNA6 |
|  |  |  |  |  | IFNA7 |
|  |  |  |  |  | IFNA8 |
|  |  |  |  |  | IFNA10 |
|  |  |  |  |  | IFNA13 |
|  |  |  |  |  | IFNA14 |
|  |  |  |  |  | IFNA16 |
|  |  |  |  |  | IFNA17 |
|  |  |  |  |  | IFNA21 |
|  |  |  |  |  | IFNG |
|  |  |  |  |  | IFNAR1 |
|  |  |  |  |  | IFNAR2 |
|  |  |  |  |  | IFNGR2 |
|  |  |  |  |  | JAK1 |
|  |  |  |  |  | JAK2 |
|  |  |  |  |  | JAK3 |
|  |  |  |  |  | TYK2 |
|  |  |  |  |  | STAT1 |
|  |  |  |  |  | STAT2 |
|  |  |  |  |  | STAT4 |
|  |  |  |  |  | STAT5A |
|  |  |  |  |  | STAT5B |
|  |  |  |  |  | STAT6 |
|  |  |  |  |  | IRF9 |
|  |  |  |  |  | EIF2AK2 |
|  |  |  |  |  | TLR3 |
|  |  |  |  |  | ZBP1 |
|  |  |  |  |  | USP21 |
|  |  |  |  |  | HSP90AA1 |
|  |  |  |  |  | HSP90AB1 |
|  |  |  |  |  | TNFAIP3 |
|  |  |  |  |  | PARP2 |
|  |  |  |  |  | PARP3 |
|  |  |  |  |  | PARP4 |
|  |  |  |  |  | H2AX |
|  |  |  |  |  | H2AC20 |
|  |  |  |  |  | H2AC12 |
|  |  |  |  |  | H2AC1 |
|  |  |  |  |  | H2AW |
|  |  |  |  |  | H2AB3 |
|  |  |  |  |  | H2AC8 |
|  |  |  |  |  | H2AC4 |
|  |  |  |  |  | MACROH2A2 |
|  |  |  |  |  | MACROH2A1 |
|  |  |  |  |  | H2AC19 |
|  |  |  |  |  | H2AJ |
|  |  |  |  |  | H2AB1 |
|  |  |  |  |  | H2AC17 |
|  |  |  |  |  | H2AC18 |
|  |  |  |  |  | H2AC11 |
|  |  |  |  |  | H2AC21 |
|  |  |  |  |  | H2AZ2 |
|  |  |  |  |  | H2AC7 |
|  |  |  |  |  | H2AZ1 |
|  |  |  |  |  | H2AC15 |
|  |  |  |  |  | H2AC6 |
|  |  |  |  |  | H2AC13 |
|  |  |  |  |  | H2AC14 |
|  |  |  |  |  | H2AC16 |
|  |  |  |  |  | H2AB2 |
|  |  |  |  |  | PPIA |
