# Supplementary material for: Identification of PANoptosis-associated genes in hepatic ischemia-reperfusion injury by integrated bioinformatics analysis and machine learning
Source: PLoS One. 2025 Dec 30;20(12):e0339651. doi: 10.1371/journal.pone.0339651 (PMC12752983; doi:10.1371/journal.pone.0339651)
Supplement: S3 Table — This table lists the forward and reverse primer sequences used for the amplification of specific genes in the study. (DOCX) [file pone.0339651.s003.docx]

# Table S3. Gene Primer Data

| **Gene** | **Forward primer (5'-3')** | **Reverse primer (5'-3')** |
| --- | --- | --- |
| IL1A | CAGGGCAGAGGAGGAGTCAAC | GAGATGGTCAATGCAACTGTAG |
| IRE3 | CCGCCTCTGAGCATCC | CCAGCTACAACGGCAGGAACC |
| CDKN1A | GAAGTCGCAGGCTAGAACAG | CAGGTCTGAATGTCAAGAGC |
| EMP1 | TGATGTGGTGGCTGTGAATTC | GTGGCTGGCGGTGAAGTTC |
| IL1B | CCTCTAACAAGCCAAG | CGATTAGAAGTCCAGCCCATAC |
| BTG3 | CGATCACTACCCTCCTGCTGT | ACATGGAATTGGGCGGAATGG |
| JUN | ACAGGCCGCACCACTTG | CCTTGATCCGCTGACACTC |
| HSPB1 | GAAGCAGGTGGTGTAGATC | ACCTGGAGGCGTGTATTTC |
| GAPDH | GCAAATTCAACGGCATCACAG | TCGGCTCCTGGAAGGTGATG |
