# Supplementary material for: Identification of PANoptosis-associated genes in hepatic ischemia-reperfusion injury by integrated bioinformatics analysis and machine learning
Source: PLoS One. 2025 Dec 30;20(12):e0339651. doi: 10.1371/journal.pone.0339651 (PMC12752983; doi:10.1371/journal.pone.0339651)
Supplement: Attachment — Submitted filename: Response to Reviewers.docx [file pone.0339651.s005.docx]

**Response to Reviewers**

Dear Editor and Reviewers,

We sincerely thank you for your meticulous review of our manuscript titled "Identification of PANoptosis-associated Genes in Hepatic Ischemia-Reperfusion Injury by Integrated Bioinformatics Analysis and Machine Learning." We greatly appreciate the time and effort you have dedicated to evaluating our work. Your constructive suggestions and comments have been invaluable in enhancing the quality of our manuscript.

We have carefully addressed all the remarks and recommendations provided by the reviewers. Your critical observations have enabled us to strengthen the relevance and clarity of our research.

Below, we provide our responses to each of the journal requirements and reviewers' comments and outline the corresponding changes made to the manuscript. All modifications have been tracked for your convenience.

Thank you once again for your support and guidance.

**To Journal Requirements:**

**Requirement 1: When submitting your revision, we need you to address these additional requirements.**

Please ensure that your manuscript meets PLOS ONE's style requirements,including those for file naming. The PLOS ONE style templates can be found at <https://journals.plos.org/plosone/s/file?id=wjVg/PLOSOne_formatting_sample_main_body.pdf> and https://journals.plos.org/plosone/s/file?id=ba62/PLOSOne_formatting_sample_title_authors_affiliations.pdf.

**Response to requirement 1 :** Thank you for your feedback regarding the style requirements for our manuscript. We have made the necessary modifications to ensure that our manuscript now meets PLOS ONE's style requirements, including proper file naming according to the provided templates.

**Requirement 2:** To comply with PLOS ONE submissions requirements, in your Methods section, please provide additional information regarding the experiments involving animals and ensure you have included details on (a) methods of sacrifice, (b) methods of anesthesia and/or analgesia, and (c) efforts to alleviate suffering.

**Response to requirement 2:** We obtained ethics committee approval (IACUC-JT-20240711-19) for our experimental design, which complies with animal research ethics:a. Method of sacrifice: Euthanasia was performed through cardiac blood collection and cervical dislocation after anesthesia with pentobarbital sodium.

b. Anesthesia was achieved through intraperitoneal administration of pentobarbital sodium (1%, 50 mg/kg). c. The depth of anesthesia in mice was carefully monitored before the procedure to ensure there was no pain.

**Requirement 3:** We note that the grant information you provided in the ‘Funding Information’ and ‘Financial Disclosure’ sections do not match. When you resubmit, please ensure that you provide the correct grant numbers for the awards you received for your study in the ‘Funding Information’ section.

**Response to requirement 3:** Thank you for bringing this to my attention. I apologize for the discrepancy in the grant information provided in the ‘Funding Information’ and ‘Financial Disclosure’ sections. I will verify the correct grant numbers and ensure that they match when I resubmit my manuscript.

**Requirement 4:** Thank you for stating in your Funding Statement:

This work was supported by the NSFC No.82360111; Xinjiang Science and Technology Department—Leading talents in technological innovation - high-level leading talents NO.2022TSYCLJ0034; State Key Laboratory for the Cause and Control of High Incidence in Central Asia Jointly Constructed by the Ministry and the Province NO.SKL-HIDCA-2023-2 and the Xinjiang Uygur Autonomous Region University research project NO.XJEDU2021I016. Please provide an amended statement that declares *all* the funding or sources of support (whether external or internal to your organization) received during this study, as detailed online in our guide for authors at http://journals.plos.org/plosone/s/submit-now. Please also include the statement “There was no additional external funding received for this study.” in your updated Funding Statement. Please include your amended Funding Statement within your cover letter. We will change the online submission form on your behalf.

**Response to requirement 4:** This work was supported by the NSFC No.82360111; Xinjiang Science and Technology Department—Leading talents in technological innovation - high-level leading talents NO.2022TSYCLJ0034; State Key Laboratory for the Cause and Control of High Incidence in Central Asia Jointly Constructed by the Ministry and the Province NO. SKL-HIDCA-2023-2; and Xinjiang Uygur Autonomous Region Graduate Innovation Program, No. XJ2024G153. Funding was provided to support the study design, the successful execution of the experiments, the purchase of experimental materials, and the payment of article publication fees. The authors associated with these grants are Tuerganaili Aji and Yingmei Shao. The funders had no role in data collection and analysis, decision to publish, or preparation of the manuscript. There was no additional external funding received for this study. We will include our amended Funding Statement within our cover letter.

**Requirement 5:** Your ethics statement should only appear in the Methods section of your manuscript. If your ethics statement is written in any section besides the Methods, please move it to the Methods section and delete it from any other section. Please ensure that your ethics statement is included in your manuscript, as the ethics statement entered into the online submission form will not be published alongside your manuscript.

**Response to requirement 5:** We confirm that the ethics statement (IACUC-JT-20240711-19) only appears in the Methods section under the "Animal and HIRI Model" subsection, and it does not appear in any other sections.

**Requirement 6:** Please include captions for your Supporting Information files at the end of your manuscript, and update any in-text citations to match accordingly. Please see our Supporting Information guidelines for more information: http://journals.plos.org/plosone/s/supporting-information.

**Response to requirement 6:** Thank you for your feedback regarding the Supporting Information files. We will ensure that captions for each Supporting Information file are included at the end of our manuscript. Additionally, we will update all in-text citations to ensure they correspond correctly to these captions.

**Requirement 7:** If the reviewer comments include a recommendation to cite specific previously published works, please review and evaluate these publications to determine whether they are relevant and should be cited. There is no requirement to cite these works unless the editor has indicated otherwise.

**Response to requirement 7:** Thank you for your guidance regarding the reviewer's comments on citing specific previously published works. We will carefully review and evaluate these recommendations to determine their relevance to our manuscript.

**To Reviewer Comments:**

**Reviewer #1**

**Comment 1 :** The use of ten machine learning algorithms and numerous bioinformatics tools, while comprehensive, may overwhelm readers and complicate the interpretation of results. A more focused methodological approach or clearer justification for the use of each algorithm would improve transparency.

**Response to comment 1 :**

We sincerely thank the reviewer for the valuable comments. We would like to clarify that our study actually employed 11 machine-learning algorithms, rather than the 10 reported in the initial submission—we mistakenly conflated two similar algorithms into one. The algorithms are: plsRglm, glmBoost, Ridge, Enet (Elastic Net), Lasso, GBM, XGBoost, SVM, Stepglm, LDA, and Naive Bayes. This correction has been updated in the revised manuscript (Methods section, subsection on machine-learning-based feature gene selection).

We highly value your observation that employing eleven machine-learning algorithms together with multiple bioinformatics tools may overwhelm readers or complicate interpretation. We recognize that while this multi-faceted strategy enhances the robustness of our findings, greater transparency can make the methodology more accessible. In response, we plan to revise the manuscript, and we will revise the workflow diagram (Figure 1) to more clearly visualize how these elements interconnect, thereby reducing any perception of overload.

Our motivation for using multiple algorithms was to further reduce dimensionality and to ensure robustness, reproducibility, and generalizability when identifying PANoptosis-related diagnostic genes across multiple independent hepatic ischemia–reperfusion injury (IRI) datasets. Each algorithm represents a distinct modeling paradigm: linear regression (Ridge, Lasso, Enet), ensemble learning (GBM, XGBoost, glmBoost), dimensionality reduction (plsRglm), probabilistic classification (Naive Bayes, LDA), margin-based optimization (SVM), and stepwise model selection (Stepglm). By employing different combinations of these diverse approaches, we minimized biases associated with any single algorithm and cross-validated predictive performance from complementary perspectives.

To enhance transparency, we have provided a dedicated explanation of the scientific basis for our integration strategy in the Methods section and explicitly state that the final gene-selection scheme is based on consensus performance—i.e., the highest mean C-index across five independent validation cohorts—rather than the standalone performance of any single algorithm. We believe this systematic framework does not add complexity; instead, it improves methodological rigor and strengthens the interpretability of the results.

We also ensured that the bioinformatics tools and machine-learning algorithms were applied in a logical sequence: gene identification → enrichment analyses (GSEA/GO/KEGG) → clustering → machine-learning screening → single-cell analysis → immune analyses (ssGSEA/CIBERSORT) → drug–target network analysis to nominate potential therapeutic agents.

**Comment 2 :** The proposed drug candidates are identified solely through network analysis, with no in vitro or in vivo efficacy testing to support their therapeutic potential.

**Response to comment 2 :**

Thank you for your insightful comment. We agree that the identification of potential drug candidates through network analysis alone is preliminary and lacks experimental validation, which limits its interpretability and strength in the current study. In response to this feedback, we have removed the drug network clustering analysis section (including related figures and discussion points) from the revised manuscript. This revision allows us to maintain focus on the core bioinformatics, machine learning, and experimental findings related to PANoptosis-associated genes in hepatic IRI, which are supported by multi-omics analysis, single-cell profiling, immune infiltration assessment, and in vivo qRT-PCR validation in a mouse model.

We believe this strengthens the overall manuscript by emphasizing robust, evidence-based insights while avoiding unsubstantiated speculation. If additional details or further revisions are needed, we are happy to address them.

**Comment 3 :** The manuscript should more explicitly address methodological and translational limitations.

**Response to comment 3 :**

Thank you for this important comment. We have substantially revised and expanded the Limitations section to more explicitly address methodological and translational constraints as follows:

This study has several important limitations that should be acknowledged.

Data heterogeneity and bioinformatics constraints. Our multi-omics integrated analysis relies on public databases with diverse sample sizes, platforms, and experimental protocols. Although batch-effect correction was applied, residual technical variation may introduce bias. Immune-cell deconvolution algorithms (CIBERSORT, ssGSEA) may not fully capture the complexity of immune populations in injured liver tissue.

Machine learning considerations. While we employed eleven algorithms and evaluated 83 combinations, the final eight-gene signature prioritizes predictive accuracy over mechanistic insight; biologically relevant genes with lower predictive power may have been excluded. Overfitting risk cannot be entirely eliminated despite nested cross-validation. External validation in independent prospective clinical cohorts is essential to confirm diagnostic utility.

Limited experimental validation. We only conducted transcriptional-level validation in a mouse IRI model via qRT-PCR and immunohistochemistry. Functional assays (gene knockdown/overexpression) to establish causal roles, protein-level quantification, and temporal dynamics were not performed. Species-specific differences may limit translatability to human hepatic IRI.

Clinical applicability. The datasets analyzed may not represent global genetic and environmental diversity. Clinical information (IRI severity, ischemic duration, patient demographics) was often incomplete or heterogeneous, precluding subgroup analyses. Prospective multi-center validation with standardized clinical annotation is needed to assess real-world applicability.

Translational gap. As a bioinformatics-driven exploratory study, this work provides a foundational framework but does not deliver ready-to-use clinical tools. Future research will include: (1) clinical validation; (2) functional mechanistic studies; (3) in vitro and in vivo pharmacological testing; and (4) development of clinically deployable diagnostic assays.

**Comment 4 :**

While the manuscript is generally well-structured, long and complex sentences—especially in the Introduction and Methods—reduce overall readability. Simplifying sentence structure and improving transitions would enhance clarity. Some technical terms are introduced without prior definition, which may hinder understanding for non-specialist readers. Certain sentences appear awkward or non-native, likely due to direct translation or complex phrasing, and would benefit from editing for clarity and conciseness.

**Response to comment 4 :**

We sincerely appreciate the reviewer's constructive feedback regarding the manuscript's readability and language clarity. We have carefully revised the entire manuscript, with particular attention to the Introduction and Methods sections, to address these concerns. The specific improvements include:

1. Sentence Structure Simplification:

We have systematically broken down long, complex sentences into shorter, more digestible units. For example:

- Original: "The inflammatory response of IRI increases the short-term risks of acute rejection, early allograft dysfunction or primary non-function, liver function failure, and even multi-organ dysfunction."
- Revised: "The inflammatory response in IRI increases short-term risks, including acute rejection, early allograft dysfunction or primary non-function, liver failure, and even multi-organ dysfunction."

2. Technical Term Definitions:

We have addressed undefined technical terms by either:

- Providing brief clarifications (e.g., expanded "MOVICS" to "Multi-Omics integration and VIsualization in Cancer Subtyping")
- Expanding abbreviations in the Methods section (e.g., "plsRglm" to "Partial Least Squares Regression for Generalized Linear Models")
- Adding contextual explanations where necessary

3. Improved Transitions and Flow:

We have enhanced logical connections between ideas and improved paragraph transitions. For instance:

- Original: "PANoptosis emerges as a recently discovered programmed cell death modality, representing a paradigmatic transformation in our understanding of cellular demise."
- Revised: "PANoptosis is a recently described programmed cell death pathway characterized by the integrated activation of apoptosis, necroptosis, and pyroptosis."

4. Language Clarity and Conciseness:

We have eliminated awkward phrasing and overly complex expressions throughout. Examples include:

- Changed "dynamically interweaving to collectively orchestrate the landscape of cellular injury" to "dynamically interact to shape cellular injury"
- Simplified "Elucidating the inflammatory mechanisms underlying hepatic IRI is critical for advancing novel therapeutic interventions" to "A deeper understanding of the inflammatory mechanisms of hepatic IRI is essential for developing therapies"

5. Enhanced Readability in Methods:

We have restructured methodological descriptions to improve clarity while maintaining scientific precision, providing more straightforward explanations of complex analytical procedures (e.g., the MOVICS clustering approach and machine learning algorithm descriptions).

These revisions have significantly improved the manuscript's accessibility for both specialist and non-specialist readers while preserving scientific rigor and accuracy.

The examples provided above represent only a portion of the revisions made. For a comprehensive view of all modifications, please refer to the revised manuscript with tracked changes (manuscript-revised).

**Reviewer #2**

**Comment :**

The research successfully integrates bioinformatics and machine learning approaches to identify eight PANoptosis-related signature genes (IER3, CDKN1A, EMP1, IL1B, BTG3, JUN, HSPB1, and IL1A) which can be potentially used as biomarker and therapeutic agent for hepatic ischemia-reperfusion injury (IRI).This submission is an original work that is methodologically sound and thoroughly documented, with experiments and analysis conducted with high level of technical rigor. The authors’ documentation on the methodology, machine learning tools and the use of available data further enhances reproducibility of this work. The procedure for validation also ensures that the research meets all applicable standards for the ethics of experimentation and research integrity. I therefore recommend it for publication

**Response to comment :**

We sincerely thank the reviewer for the positive evaluation and recommendation for publication. We appreciate the recognition of our methodological approach and the potential clinical relevance of this study.We hope that this work may contribute to the understanding of PANoptosis-related mechanisms in hepatic IRI and provide a basis for future research in this field.We thank the reviewer for their time and thoughtful evaluation of our manuscript.

**Reviewer #3**

**Comment (a-e) :**

(a) On page 21, the authors note that, "A drug-gene target network was established using eight characteristic genes…" (only 7 are listed) which was based upon the data revealed by the study. Using this network, the authors screened JUN, HDAC2, NR3C1 and ESR1 for the therapeutic potential of several drugs. The rationale for choosing these targets (and drugs) is not perfectly clear and should be explained further.

(b) A legend for Supplementary Figure 1 is required.

(c) The legend should describe the relationships (line connectors) among drugs, targets and the characteristic genes (HSPB1, CDKN1, ILA and ILB) in Fig. S1A. What does the reticle displayed in the upper left (0 to 15) indicate?

(d) Similar information explaining the drug relationships in Fig. S1B is required.

(e) Complete explanations of the colors and groupings depicted in Fig. S1C must likewise be included.

**Response to comment (a-e) :**

We sincerely thank the reviewer for these detailed and constructive comments regarding the drug–gene target network analysis and Supplementary Figure 1. We fully agree that the rationale for drug/target selection and the interpretation of the corresponding network require clearer explanation and, more importantly, experimental validation to support the therapeutic relevance of these predictions.

Upon further reflection, we realized that this section, which was originally included as supplementary exploratory content, was not analyzed in sufficient depth and lacked experimental validation. Although the preliminary results appear interesting, the current evidence is insufficient to justify strong biological or therapeutic conclusions. Moreover, inclusion of this exploratory analysis may detract from the main aim and focus of our study.

Therefore, to strengthen the scientific rigor and clarity of the revised manuscript, we have “removed the entire drug–gene network analysis section (including Supplementary Figure 1 and related text)”. The revised version now focuses on the validated bioinformatics findings and experimental results regarding PANoptosis-associated gene signatures in hepatic ischemia–reperfusion injury (IRI), which are supported by multi-omics integration, single-cell RNA sequencing analysis, immune infiltration profiling, and in vivo qRT-PCR validation.

We believe this revision enhances the focus, coherence, and credibility of the study while avoiding overinterpretation of exploratory computational results. We are grateful to the reviewer for the insightful suggestions, which have helped us clarify the manuscript’s scope and strengthen its overall scientific quality.
