# Supplementary material for: Identification of PANoptosis-associated genes in hepatic ischemia-reperfusion injury by integrated bioinformatics analysis and machine learning
Source: PLoS One. 2025 Dec 30;20(12):e0339651. doi: 10.1371/journal.pone.0339651 (PMC12752983; doi:10.1371/journal.pone.0339651)
Supplement: Attachment — Submitted filename: Response to Reviewers_2.docx [file pone.0339651.s006.docx]

**Response to Reviewers**

Dear Editor and Reviewers,

We sincerely thank you for the positive feedback on our revised manuscript (Identification of PANoptosis-associated Genes in Hepatic Ischemia-Reperfusion Injury by Integrated Bioinformatics Analysis and Machine Learning, PONE-D-25-02077R1) and for acknowledging that our previous revisions adequately addressed the initial concerns.

In this revision, we have focused on the minor editorial comments provided by Reviewer #3 (Dr. John M. Aletta) to further enhance the clarity and consistency of the manuscript.

We have made the necessary corrections to the figure legends and abbreviations as requested. All modifications have been tracked for your convenience.

Thank you once again for your support and guidance. Wishing you a wonderful Thanksgiving!

**To Journal Requirements:**

**Requirement :** If the reviewer comments include a recommendation to cite specific previously published works, please review and evaluate these publications to determine whether they are relevant and should be cited. There is no requirement to cite these works unless the editor has indicated otherwise.

Please review your reference list to ensure that it is complete and correct. If you have cited papers that have been retracted, please include the rationale for doing so in the manuscript text, or remove these references and replace them with relevant current references. Any changes to the reference list should be mentioned in the rebuttal letter that accompanies your revised manuscript. If you need to cite a retracted article, indicate the article’s retracted status in the References list and also include a citation and full reference for the retraction notice.

**Response to requirement :** We confirm that the reviewers acted with high professional standards and did not recommend citing any specific previously published works or their own publications.

Regarding the reference list, we have managed all citations using EndNote to ensure accuracy and completeness. Furthermore, we have rigorously screened our bibliography for retracted articles by cross-referencing multiple authoritative databases, including PubMed, Web of Science, and the Retraction Watch database. As of November 27, 2025, no retracted articles have been identified in our reference list.

**To Reviewer Comments:**

**Reviewer #3**

**Comment 1 :** Figure 1, Overview of Study Workflow, has no legend. It is recommended that a legend with a brief description of the study workflow including spelling out the full meaning of key acronyms should be added.

**Response to comment 1 :** Thank you for your valuable suggestion. We agree that a detailed legend is essential for clarifying the study design. We have added a figure legend to Figure 1 in the revised manuscript. This legend provides a step-by-step description of the workflow, from data collection (GEO and KEGG) and the identification of DE-PANRGs, to the application of multi-omics clustering algorithms and machine learning models. Additionally, we have spelled out all key acronyms used in the figure to ensure clarity for readers.

**Comment 2 :** The principal acronym for hepatic ischemia-reperfusion injury should be consistent throughout the manuscript. Not IRI some times and HIRI others.

**Response to comment 2 :**

We sincerely thank the reviewer for their attention to detail regarding the terminology. We agree that consistency is crucial. After careful review, we have decided to standardize the terminology to "hepatic IRI" throughout the manuscript when referring to liver-specific injury, rather than using the acronym "HIRI". This ensures precision while maintaining readability. We have corrected all inconsistent instances (such as "HIRI", "liver IRI", or mixed usage) to "hepatic IRI". The acronym "IRI" is now used exclusively when discussing general ischemia-reperfusion injury mechanisms not specific to the liver.
